# The MMP-9 -1562 C/T Polymorphism in the Presence of Metabolic Syndrome Increases the Risk of Clinical Events in Patients with Coronary Artery Disease

**DOI:** 10.1371/journal.pone.0106816

**Published:** 2014-09-05

**Authors:** Trine B. Opstad, Harald Arnesen, Alf Å. Pettersen, Ingebjørg Seljeflot

**Affiliations:** 1 Center for Clinical Heart Research, Department of Cardiology, Oslo University Hospital Ullevål, Oslo, Norway; 2 Center for Heart Failure Research, Oslo University Hospital, Oslo, Norway; 3 Faculty of Medicine, University of Oslo, Oslo, Norway; Washington Hospital Center, United States of America

## Abstract

**Background and Objectives:**

Elevated levels of matrix metalloproteinase (MMP)-9 have been associated with the metabolic syndrome (MetS) and cardiovascular events. The MMP-9 −1562 C/T polymorphism has furthermore been shown as a risk factor for coronary artery disease (CAD). The non-favourable cardiometabolic state in MetS may increase the risk. We aimed to investigate the influence of MMP-9 −1562 C/T polymorphism in subjects with CAD and MetS.

**Methods:**

Patients (n = 1000) with verified CAD stratified in Mets +/− (n = 244/756), were analyzed for the MMP-9 −1562 C/T polymorphism and related to clinical events after 2 years follow-up. Serum levels of total MMP-9 and tissue inhibitor of matrix metalloproteinases (TIMP)-1were analyzed in all, whereas MMP-9 activity, extracellular matrix metalloproteinase inducer (EMMPRIN), and expression of the two genes were analyzed in a subset of 240 randomly selected patients.

**Results:**

Totally, 106 clinical endpoints were recorded. In MetS; the T-allele associated with 5.5 fold increase in event rate (p<0.0001), increased with number of MetS components, a 117% increase in total MMP-9 levels (TT homozygous, p = 0.05), significantly higher total- and endogenous active MMP-9 and TIMP-1 levels (p<0.01 all), and EMMPRIN was inversely correlated with pro- and endogenous active MMP-9 (p<0.05, both). In non-MetS; the T-allele was not associated with new events, nor higher MMP-9 levels. EMMPRIN was significantly correlated with total MMP-9 and TIMP-1 (p<0.01, both) and the two genes were inter-correlated (p<0.001).

**Conclusion:**

In CAD patients with MetS, the MMP-9 T-allele increased the risk of clinical events, probably mediated through elevated MMP-9 levels and altered MMP-9 regulation.

## Introduction

Atherosclerosis is the main cause of coronary artery disease (CAD) and the process starts early in life in a progressive interaction between environmental and genetic factors. The metabolic syndrome (MetS), often present in CAD, is defined as a clustering of risk factors including hypertension, dyslipidaemia, hyperglycaemia and obesity, in which both environmental and genetic factors are involved. The pathophysiology of MetS involves a pro-inflammatory state, partly mediated by the production of inflammatory cytokines, which in turn may induce expression of matrix metalloproteinases (MMPs), including MMP-9, and other cytokines [1]. MetS and MMPs are thus thought to play an important role in the pathogenesis of atherosclerotic cardiovascular disease (CVD), both individually and through interaction [1], [2].

MMPs are endopeptidases that degrade extracellular matrix [3]. The MMPs are synthesized and released as pro-proteins which need to be cleaved to become biological active enzymes. MMP-9 has especially been related to plaque progression and rupture and was reported to be highly expressed in vulnerable region of atherosclerotic plaques [4]. Elevated circulating MMP-9 levels have been observed in CAD and hypertension (HT), and have also been associated with cardiovascular events and mortality [5]–[9]. Elevated MMP-9 levels and altered MMP-9 expression have furthermore been associated with MetS [1], [10], [11].

EMMPRIN (extracellular matrix metalloproteinase inducer) is an inducer of MMP-9, also known as CD 147 and Basigin. EMMPRIN is a transmembrane glycoprotein and a member of the immunoglobulin family [12]. Enhanced expression of EMMPRIN was shown on the surface of circulating monocytes in acute myocardial infarction (AMI), compared to stable angina patients, and the expression was associated with increased plasma MMP-9 activity [12]. The authors suggested that EMMPRIN may be involved in both expression and release of MMP-9 and to be a key regulator of MMP-9 activity in CVD. Another study reported a possible role of EMMPRIN in diabetic atherosclerotic complications [13]. EMMPRIN has been reported to be expressed in atherosclerotic plaques, often co-localized with monocytes and macrophages, and with MMP-9 and the inhibitor of MMP-9; TIMP (tissue inhibitor of matrix metalloproteinase) -1 [14]. The levels of TIMP-1 have also been reported to be elevated in MetS [11], whereas the role of EMMPRIN is to our knowledge unknown.

We and others have previously shown that certain MMP-9 genetic variants modify circulating levels of the protein [9], [15]. The MMP-9 −1562 C/T polymorphism is located in the MMP-9 gene promoter, thus exposed to environmental or epigenetic conditions that may induce MMP-9 expression. The T-allele has been shown to induce higher promoter activity and to increase expression of MMP-9, mediated by reduced binding of a transcription-repressor protein to the T-allele [16]. The genetic variant has been widely investigated and the MMP-9 -1562 T-allele has been associated with higher circulating MMP-9 levels, CAD severity, MI, arterial stiffness and worse prognosis in CVD [9], [15]–[18]. Recently a meta-analysis concluded that the MMP-9 -1562 C/T polymorphism is a risk factor for CHD [19]. The T-alleles' effect on circulating MMP-9 levels exclusively in obese and lack of such influence in lean people has further been reported [20], [21]. Together, these observations highlight a possible predisposition of coronary atherosclerosis in MMP-9 −1562 T-allele carriers and the non-favourable cardiometabolic state in MetS may further increase this risk.

We have performed an association study on the MMP-9 −1562 C/T polymorphism in stable CAD patients stratified for the presence or absence of MetS, in relation to clinical endpoints. We hypothesized that the additional presence of MetS increases the genetic risk of new clinical events. To explore a potential altered regulation of MMP-9 in MetS, circulating MMP-9, TIMP-1 and EMMPRIN, as well as gene expression of MMP-9 and EMMPRIN, were further assessed.

We could show that the MMP-9 -1562 C/T polymorphism in presence of MetS increased the risk of new clinical events in our CAD population. An altered MMP-9 regulation in MetS was additionally observed.

## Materials and Methods

### Study population

Patients with angiographically verified stable CAD (n = 1000, median age 62 years, 22% women, 97% of Western European descent) enrolled in the ASCET study (ASpirin non-responsiveness and Clopidogrel Endpoint Trial) [22], [23] were investigated.

The presence of MetS +/− was recorded in 244/756 patients. MetS was defined by modified NCEP criteria [24]. To be diagnosed with MetS, a threshold value in at least three of the following criteria needed to be present: 1) abdominal obesity: BMI ≥30 kg/m^2^ 2) elevated triglycerides: ≥1.69 mmol/L 3) low HDL-cholesterol: <1.04 mmol/L in male and <1.29 mmol/L in female 4) elevated blood pressure: ≥130/85 mm Hg or diagnosed or treated HT and 5) elevated fasting glucose: ≥6.1 mmol/L.

Patients were followed for a minimum of 2 years and the primary end point included the first event of a composite of nonfatal AMI, unstable angina pectoris, stroke and all-cause mortality. Relatedness in the population was <1%.

Frequency of the MMP-9 −1562 polymorphism and circulating total MMP-9 and TIMP-1 were investigated in the entire population. In a subpopulation of 240 randomly selected patients, circulating EMMPRIN, MMP-9 activity and expression of the two genes were analyzed to explore regulatory mechanisms.

The study was performed according to the Declaration of Helsinki. Evaluation of end points was performed by an end point committee without access to the laboratory data. The ASCET trial is registered at clinicaltrials.gov, with the identification number NCT00222261.

### Ethics Statement

The study was approved by The Regional Committee of Medical Research Ethics in South-Eastern Norway, and all subjects gave their written informed consent to participate.

### Laboratory analysis

In fasting conditions between 8.00–10.30 a.m., and without intake of any medications, blood samples were collected at entrance into the ASCET trial. Serum was prepared by centrifugation within 1 hour at 2.500×g in 10 minutes for determination of MMP-9, EMMPRIN, TIMP-1, and routine analyses. EDTA blood and PAXgene Blood RNA tubes (PreAnalytix, Qiagen GmbH, Germany) were collected for DNA extraction and RNA isolation, respectively. All materials were kept frozen at −80°C until further preparation and analysis. Circulating levels of total MMP-9 and TIMP-1 were determined in all patients (n = 1000), whereas EMMPRIN and MMP-9 activity (pro-protein and endogenous active protein) were analyzed in the subset of 240 patients (MetS +/−, n = 68/172), in which the endogenous active MMP-9 protein was detectable in 104 due to low sensitivity of the assay (MetS +/−, n = 33/71). MMP-9 activity was analyzed by the Fluorokine E quantitative assay (R&D Systems, Europe, Abingdon, Oxon, UK) designed to measure both the endogenous active enzyme and the pro enzyme that can be activated by aminophenylmercurie acetate during the assay procedure, both assumed to be biologically important. MMP-9 (total amount), TIMP-1 and EMMPRIN concentrations were measured by convential ELISA (R&D Systems). The inter-CVs for the MMP-9 activity, MMP-9, TIMP-1 and EMMPRIN assays were 7.5%, 7.3%, 4.4% and 8.0%, respectively.

DNA was isolated from whole blood using a MagNA Pure LC DNA Isolation kit (Roche Diagnostics, GmbH, Mannheim, Germany). DNA purity and quantity were tested on the NanoDrop, ND-1000 (Saveen Werner, Sweden). The MMP-9 −1562 C/T polymorphism (rs3918242) was genotyped by Real-Time PCR using primers and probes synthesized by TIB MOLBIOL (D-12103 Berlin, Germany), and Light Cycler FastStart DNA Master Hybridization Probes kit (Roche Diagnostics), previously described in details [15]. The MMP-9 −1562 C/T polymorphism was successfully determined in 996 samples. Genotype calling was automatically applied in the assay, with 95% confidence. About 5% of the samples were repeated, with 100% concordance.

In the subset of 240 patients, total RNA in circulating leukocytes was extracted from the PAXgene Blood RNA tubes by use of the PAXgene Blood RNA kit (PreAnalytix, Qiagen GmbH, Germany), with an extra cleaning step (RNaseasy MinElute Cleanup kit, Qiagen). RNA purity and quantity were tested on the NanoDrop, ND-1000. Total RNA was reversely transcribed in a total volume of 20 µl, using the Omniscript RT Kit (Qiagen), Oligos (dTs) and Rnase Inhibitor (Applied Biosystems, Foster City, CA, USA) for MMP-9 analysis and the qScript cDNA Super Mix (Qiagen) for EMMPRIN analysis. MMP-9 and EMMPRIN mRNA levels were determined by real-time PCR on the ABI Prism 7900 HT Sequence Detection System and ViiA 7 instruments, respectively, with TaqMan Gene Expression Assays (MMP-9, HS00234579_m1 and EMMPRIN, Hs00936295_m1) and TaqMan Fast Universal PCR Master Mix (2X), No AmpErase UNG (Applied Biosystems). The MMP-9 and EMMPRIN mRNA levels were normalized to β-2-microglobulin (HS99999907_m1, Applied Biosystems) and fold expression was determined as previously described [25]. The MMP-9 and EMMPRIN gene expression analyses were successfully determined in 240 and 238 samples, respectively.

### Statistical analysis

As most of the measured variables were skewely distributed, non- parametric tests were applied. For group comparisons, the Mann-Whitney and Kruskal Wallis tests, when appropriate, were used for continuous data and the χ^2^ test for categorical data. The association between circulating markers and MetS was analyzed by linear regression using log transformed data, when appropriate, whereas the MMP-9 genotype-association with MetS was performed by logistic regression, both models adjusted for age and gender. Other baseline characteristics, including MetS criteria, were not differently distributed between MetS patients suffering new clinical events or not, thus not corrected for in the regression models.

Spearman's rho was used in the correlation analysis. The Hardy-Weinberg equilibrium (HWE) was tested using the χ^2^ test. All statistical analyses were performed by SPSS 19.0 (SPSS Inc., Chicago, Illinois, USA). A two-tailed probability test of 0.05 or less was considered statistically significant.

## Results

After two years, a total of 106 composite clinical endpoints were recorded. The number was not differently distributed in subjects with and without MetS (10.2%, n = 25 versus 10.7%, n = 81, *p* = 0.84).

Baseline characteristics in patients according to having MetS or not, and in MetS patients suffering clinical event or not, are presented in Table 1, showing expected differences between MetS + and Mets – patients. Distribution of baseline characteristics in the subpopulation of 240 patients according to MetS +/− was similar to the data presented for the total population. The frequency of the MMP-9 −1562 C/T polymorphism according to the presence or absence of MetS is shown in Table 2. No statistically significant difference between the groups was observed. The influence of the MMP-9 −1562 C/T polymorphism on circulating levels in the total cohort has previously been reported on [15], showing the highest MMP-9 levels in MMP-9 T-allele carriers. The MMP-9 genotypes were in HWE and the observed minor allele frequency is in line with previous reports.

**Table 1:** Baseline characteristics in the total population with and without the presence of MetS and in MetS patients according to clinical endpoints.

	Total population (n = 1000)			MetS patients (n = 244)		
	MetS + (n = 244)	MetS - (n = 756)	*p* a	Endpoint + (n = 25)	Endpoint - (n = 219)	*p* b
Age (years, mean (range))	61 (39–81)	63 (36–80)	0.004	63 (54–76)	61 (39–81)	ns
Men/Women n (%)	192/52 (79/21)	590/166 (78/22)	ns	18/7 (72/28)	174/45 (79.5/20.5)	ns
Type 2 Diabetes n (%)	127 (52)	73 (10)	<0.001	13 (52)	114 (52)	ns
Previous MI n (%)	117 (48)	320 (42)	0.124	14 (56)	103 (47)	ns
Hypertension n (%)	203 (83)	350 (47)	<0.001	22 (88)	181 (83)	ns
Current smokers n (%)	54 (22)	148 (20)	ns	7 (28)	47 (22)	ns
BMI (kg/m^2^)^c^	30.4 (28.0, 32.8)	26.2 (24.3, 28.1)	<0.001	30.4 (26.9, 32.9)	30.4 (28.1, 32.8)	ns
Total cholesterol (mmol/L)	4.6 (0.9)	4.5 (1.0)	ns	4.3	4.6	ns
HDL cholesterol (mmol/L)	1.0 (0.3)	1.4 (0.4)	<0.001	1.1	1.0	ns
Triglycerides (mmol/L)^c^	1.95 (1.65, 2.59)	1.13 (0.87, 1.55)	<0.001	1.99	1.94	ns
Fasting glucose (mmol/L)	7.4 (2.4)	5.6 (1.4)	<0.001	7.3	7.4	ns

Values are mean (SD) or number (proportions) if not otherwise stated. SD: standard deviation, MI: myocardial infarction, BMI: body mass index, HDL: high density lipoprotein.

a *p*-values refer to differences between patients with and without Mets,

b p-values refer to differences between MetS patients with and without clinical endpoint.

c Median levels (25, 75 percentiles).

**Table 2:** Frequency of the MMP-9 −1562 C/T polymorphism as related to MetS +/−.

	Total (%)	MetS + (%)	MetS – (%)	*p*
MMP-9 −1562	n = 996^a^	n = 244^a^	n = 751^a^	
CC	756 (75.9)	182 (74.6)	573 (76.3)	
CT	225 (22.6)	60 (24.6)	165 (22.0)	
TT	15 (1,5)	2 (0.8)	13 (1.7)	
T-allele frequency	0.128	0.131	0.127	ns

a Number refers to subjects with available genotypes, and MetS status.

*p*-value refers to difference in frequency of the MMP-9 −1562 T-allele in subjects with and without MetS.

### The MMP-9 −1562 C/T polymorphism, MetS and clinical outcome

**Figure 1:**
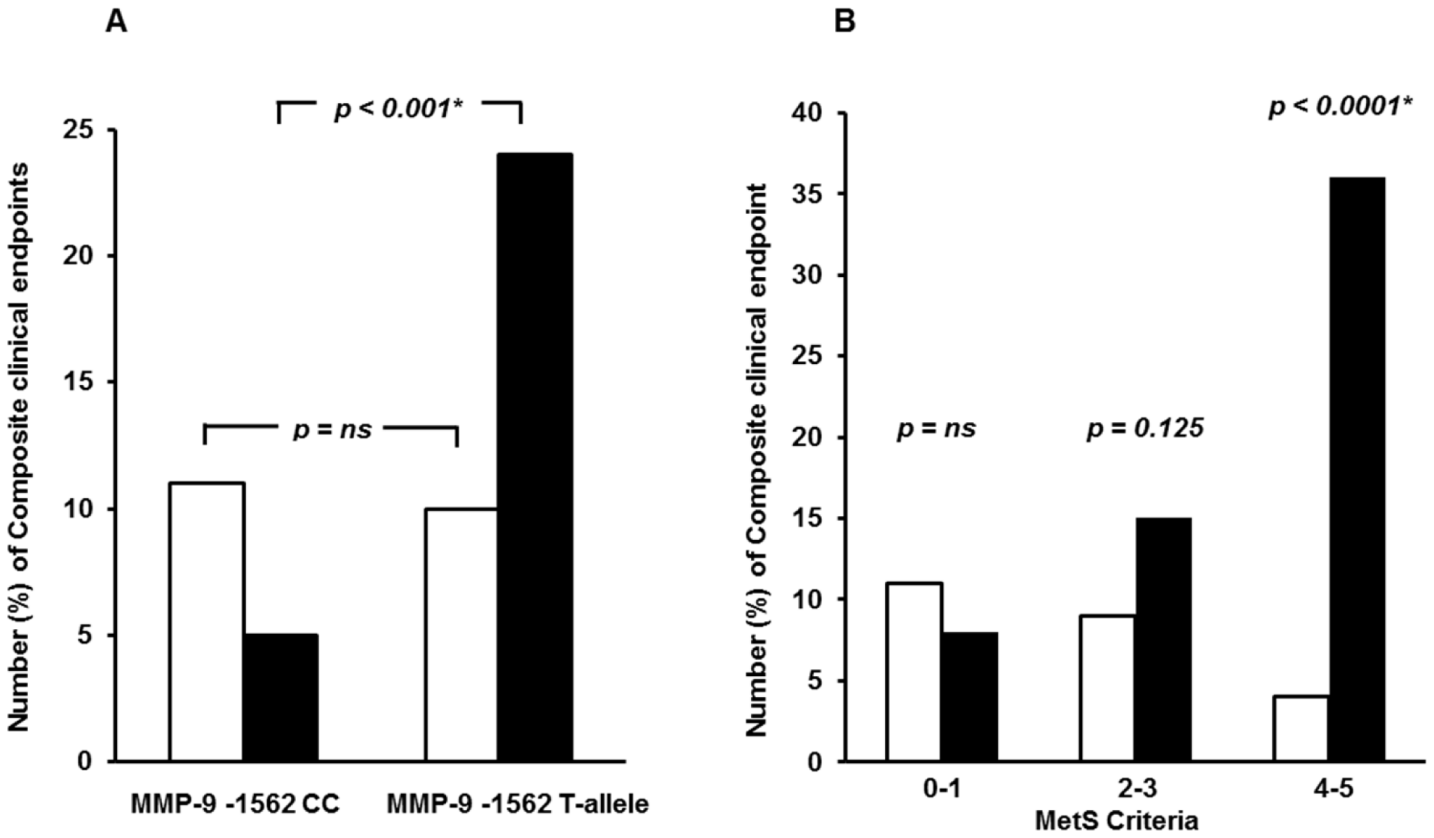
Influence of the MMP-9 −1562 C/T polymorphism on clinical events in CAD patients with MetS. **A. Number (%) of Composite clinical endpoints in CAD patients as related to the MMP-9 −1562 C/T polymorphism and MetS +/−.** White bars: MetS – (CC n = 63, T-allele n = 18), black bars: MetS + (CC n = 10, T-allele n = 15) *p*-values refer to risk of endpoints in T-allele carriers as compared to the CC genotype. ^*^ adjusted for age and gender. **B. Number (%) of Composite clinical endpoints in CAD patients as related to the MMP-9 −1562 C/T polymorphism and number of MetS criteria.** White bars: CC genotype, black bars: T-allele. *p*-values refer to risk of endpoints in T-allele carriers as compared to CC genotype when MetS criteria are categorized into 3 groups; 0 and 1 MetS criteria (CC n = 46, T-allele n = 9), 2 and 3 MetS criteria (CC n = 24, T-allele n = 14), and 4 and 5 Mets criteria (CC n = 3, CT n = 10). ^*^ adjusted for age and gender.

The risk profile stratified according to MetS +/− and the MMP-9 −1562 C/T polymorphism is shown in Figure 1A. In patients with MetS, the MMP-9 T-allele associated with significantly increased risk of new events compared to the MMP-9 CC genotype [OR = 5.5 (95% confidence interval (CI): 2.3, 13.2), *p*<0.001, adjusted]. In non-MetS patients, the T-allele had no significant influence on risk compared to the wild-type (*p* = 0.74).When categorizing all patients according to number of MetS criteria into 3 groups (0 and 1 MetS criteria, 2 and 3 MetS criteria, and 4 and 5 Mets criteria), a significant increase in number of clinical endpoints was observed in T-allele carriers through groups (*p* for trend  = 0.001). The difference in number of endpoints between CC homozygous and T-allele carriers in the group with 4 or 5 MetS criteria was highly significant [OR = 13.3 (95% CI: 3.3, 53.5), *p*<0.001, adjusted]. (Figure 1B).

### Circulating markers, MetS and clinical outcome

Circulating levels of total-, endogenous active MMP-9 and TIMP-1 were significantly higher in MetS subjects compared to non-Mets subjects (adjusted *p*<0.05, all), whereas levels of pro-MMP-9 and EMMPRIN were not (*p* = 0.15 and *p* = 0.64, respectively) (Table 3). Total MMP-9 levels increased significantly with increasing number of MetS Criteria categorized in the 3 groups (*p* for trend  = 0.031). However, MMP-9 levels were not significantly correlated to any of the MetS components, except for very weak correlation to triglycerides (r = 0.098), independent of the presence of MetS or not.

**Table 3:** Circulating levels of MMP-9, TIMP-1 and EMMPRIN as related to MetS +/−.

	MetS – (756)	MetS + (244)
MMP-9 ng/mL (n = 1000)	230 (160, 340)	251 (183, 405)*
MMP-9 activity ng/mL, pro-protein (n = 240)	204 (130, 335)	255 (132, 435)
MMP-9 activity ng/mL, endogenous (n = 104)	23 (12, 42)	40 (20, 72)*
TIMP-1 ng/mL (n = 1000)	168 (149, 190)	176 (154, 202)*
EMMPRIN pg/mL (n = 240)	3353 (3004, 3797)	3496 (2828, 3883)

Levels are median (25, 75 percentiles).

^*^ *p*<0.05 refers to difference in circulating levels of the markers between MetS +/−, adjusted for age and gender.

**Figure 2:**
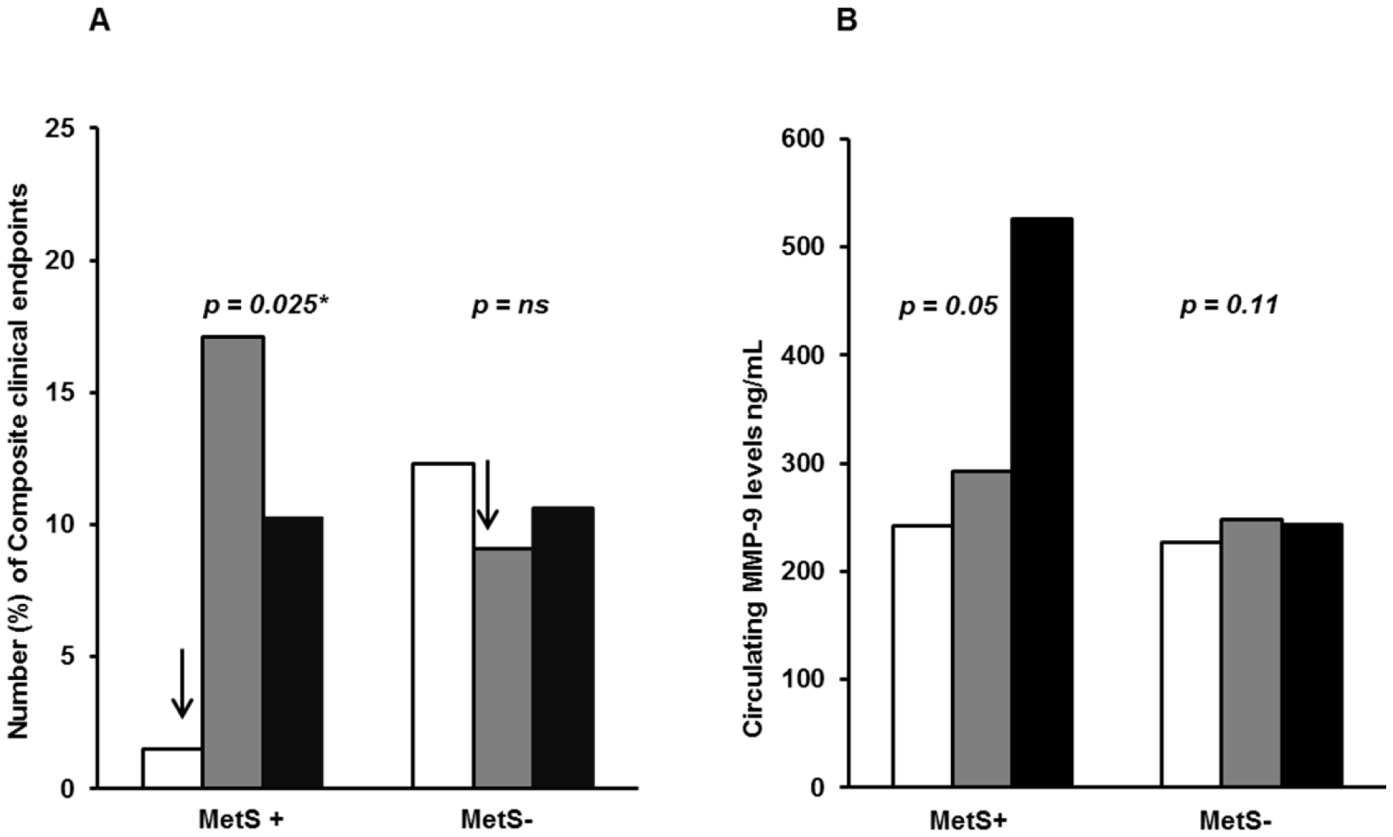
Circulating MMP-9 levels according to clinical events and the MMP-9 −1562 C/T polymorphism. **A. Tertiles of total MMP-9 levels as related to Composite clinical endpoints and MetS +/−.** White bars: Tertile 1, grey bars: Tertile 2, black bars: Tertile 3. 33percentile  = 186.9 ng/mL, 66 percentiles  = 305.8 ng/mL. The number in each Tertile group (1-2-3) in MetS; 1-14-10, in non-MetS; 33-23-25. *p*-values refer to the comparison of groups dichotomized between the lowest and the two upper tertiles. ^*^ adjusted for age and gender. **B. Circulating levels of total MMP-9 according to different MMP-9 −1562 C/T genotypes in MetS +/−.** White bars: CC genotype, grey bars: CT genotype, black bars: TT genotype. The number in each genotype group CC-CT-TT in MetS; 182- 60- 2, in non-MetS; 573-165-13. *p*-values refer to difference in circulating MMP-9 levels according to MMP-9 -1562 genotypes, Kruskal Wallis test.

As previously reported on, circulating MMP-9 was not elevated in patients suffering new clinical events [26]. When dividing total MMP-9 levels into tertiles, a significant difference in number of clinical endpoints was observed through tertiles in the MetS group (Figure 2A). MMP-9 levels in the two upper tertiles were strongly associated with incidence of new clinical events as compared to the lowest tertile [OR = 9.8 (95% CI: 1.3, 74.6), adjusted *p* = 0.025]. No significant association through tertiles was observed in the non-MetS group (*p* = 0.49).

The influence of the MMP-9 −1562 polymorphism according to number of T-alleles (CC-CT-TT) on circulating MMP-9 levels deviated between MetS and non-Mets. The increase in number of T-allele associated with significant increase in MMP-9 levels in MetS (*p* = 0.05), whereas no significant association was observed in non-Mets (*p* = 0.11) (Figure 2B). The pro- and endogenous active MMP-9 protein were not significantly influenced by the different MMP-9 genotypes, as previously reported [15], independent of the presence of MetS or not.

### Correlations between the circulating markers

In the cohort of 240 subjects, total MMP-9 correlated to MMP-9 pro-protein activity (r = 0.75), endogenous MMP-9 activity (r = 0.35) and to TIMP-1 (r = 0.18), the latter also correlated to EMMPRIN (r = 0.27), (*p*<0.01, all). Comparable correlations between the different MMP-9 pools existed when the cohort was stratified in MetS +/−.

EMMPRIN was inversely correlated to MMP-9 activity in MetS subjects [pro-protein activity (n = 68) r = −0.28, and endogenous activity (n = 33) r = −0.38, p<0.05, both]. In non-Mets subjects, EMMPRIN was positively correlated with total MMP-9 (r = 0.21) and TIMP-1 (r = 0.32), *p*<0.01, both.

According to the MMP-9 -1562 C/T polymorphism in this subset, similar correlations were observed in CC homozygous (n = 183). In MMP-9 −1562 T-allele carriers (n = 57), the correlations between the variables in the MetS and non-Mets group were no longer statistically significant, except for a significant correlation between EMMPRIN and TIMP-1 in MetS subjects (n = 15, r = 0.59, *p*<0.05).

### MMP-9 and EMMPRIN gene expression in circulating leukocytes

Expression of the MMP-9 and EMMPRIN genes in the 240 cohort were not associated with MetS, and did not correlate significantly to any of the circulating MMP-9 pools or to EMMPRIN, respectively. However, in MMP-9 −1562 T-allele carriers, expression of the MMP-9 gene correlated significantly to the endogenous active MMP-9 protein (n = 24, r = 0.47, p<0.05). Expression of the two genes inter-correlated significantly, only in non-Mets subjects (r = 0.31, p<0.001), whereas EMMPRIN gene expression correlated inversely to circulating MMP-9 especially in MetS subjects (r = −0.30, p = 0.01).

## Discussion

In the present study on stable CAD patients, the main results was that the MMP-9 −1562 C/T polymorphism modified the risk of new clinical events in patients presenting with Mets, partly mediated through altered MMP-9 regulation. MetS alone did not increase the risk.

We have previously shown that the MMP-9 −1562 C/T polymorphism tended to associate with new clinical events in this population [26]. The observed five-fold increased risk observed in T-allele carriers *with* MetS compared to CC homozygous in the present investigation, highlights a potential role of this polymorphism in certain high-risk patients. Conditions that induce MMP-9 expression may interact with the MMP-9 −1562 C/T genetic variant, as the polymorphism is located in the transcription factor-binding site in the promoter. Zhang et al reported that the MMP-9 −1562 T-allele, shown to induce two-fold higher promoter activity due to weaker binding of a repressor protein to the T-allele, was weakly associated with more severe atherosclerosis in CAD patients [16]. The additional risk-profile in MetS subjects seems synergistically to increase the risk of new events in our CAD population, as we observed an increase in number of clinical endpoints with increasing number of MetS Criteria in T-allele carriers. In the MetS group, MMP-9 levels in the two upper tertiles were strongly associated with incidence of new clinical events. However, as visualized in Figure 2A, patients with MetS being in the second tertile of MMP-9 levels showed higher incidence of endpoints as compared to MetS patients in third tertile. The impact of the different MetS components may have influenced this observation, but due to the low number of endpoints, this has not been further explored. The MMP-9 polymorphism appears to interfere differently in patients with and without MetS, as the variant allele induced significantly higher levels of MMP-9 in MetS subjects as compared to non-MetS subjects. *How* the gene is activated seems to be essential, as the observed altered protein level in T-allele carriers appears to be potentiated in patients with higher degree of metabolic-related disease burden.

Regulation of MMP-9 by EMMPRIN seems also to be different according to the presence of MetS, as MMP-9 levels appear to be regulated through negative feedback mechanisms of EMMPRIN in MetS, maybe in an attempt to diminish augmented MMP-9 protein expression, release and activity in Mets subjects. MMP-9 and EMMPRIN gene- and protein levels showed similar pattern in non-Mets subjects, which may be indicative of a more regular induction of MMP-9 in patients without this metabolic disorder.

The observed correlation between MMP-9 and TIMP-1also confirms TIMP-1s' inhibitory role in MMP-9 regulation. The significant correlation between EMMPRIN and TIMP-1, especially in MetS T-allele carriers, may indicate an increased common inhibitory regulation when MMP-9 is particularly raised.

Another observation in the present study was the significant correlation between MMP-9 gene expression and the endogenous active MMP-9 protein in T-allele carriers, suggesting that the T-allele is more prone to induced *de-novo* synthesis resulting in more endogenous active protein. The MMP-9 pro-protein may thus reflect a constitutive gene-expression, which presumably is stored, released and activated upon increased request. The endogenous active MMP protein, and not the MMP-9 pro-protein, was observed significantly elevated in MetS subjects, which may support our assumptions.

In a previous study, we have shown that upon whole-blood LPS stimulation, MMP-9 was significantly released into the plasma-pool with simultaneously no increase in *de novo* synthesis of MMP-9 [15], also reported by others [27]. We could also demonstrate that MMP-9 levels were higher and EMMPRIN levels lower in the stimulated samples from Mets patients compared to non-MetS patients (data not shown). Our observations in such an exposed situation may support the suggested negative feed-back regulation of MMP-9 by EMMPRIN in MetS subjects.

The limitation in our study is the low number of clinical endpoints, and not exclusively CV endpoints, and also a rather short follow–up time. Thus, the results from the study are to a certain extent hypothesis generating. It should also been emphasized that we have used the NCEP criteria for the definition of MetS. The use of other criteria might have influenced the results.

In conclusion, we have observed a 5-fold increase in risk of clinical events in T-allele carriers of the MMP-9 -1562 C/T polymorphism in CAD patients with MetS, probably mediated through altered MMP-9 regulation. Our observations highlight the impact of the environment on the MMP-9 gene and how its expression apparently is induced and regulated by EMMPRIN and TIMP-1.
